# Peer evaluation and feedback for invasive medical procedures: a systematic review

**DOI:** 10.1186/s12909-022-03652-9

**Published:** 2022-07-29

**Authors:** Theresa Thai, Diana K. N. Louden, Rosemary Adamson, Jason A. Dominitz, Jacob A. Doll

**Affiliations:** 1 University of Washington, Seattle, WA USA; 2 VA Puget Sound Health Care System, Seattle, WA USA; 3 National Gastroenterology and Hepatology Program, Veterans Affairs Administration, Washington, DC USA

**Keywords:** Feedback, Quality Improvement, Evaluation, Procedures, Peer Review, Endoscopy, Percutaneous Coronary Intervention, Bronchoscopy

## Abstract

**Background:**

There is significant variability in the performance and outcomes of invasive medical procedures such as percutaneous coronary intervention, endoscopy, and bronchoscopy. Peer evaluation is a common mechanism for assessment of clinician performance and care quality, and may be ideally suited for the evaluation of medical procedures. We therefore sought to perform a systematic review to identify and characterize peer evaluation tools for practicing clinicians, assess evidence supporting the validity of peer evaluation, and describe best practices of peer evaluation programs across multiple invasive medical procedures.

**Methods:**

A systematic search of Medline and Embase (through September 7, 2021) was conducted to identify studies of peer evaluation and feedback relating to procedures in the field of internal medicine and related subspecialties. The methodological quality of the studies was assessed. Data were extracted on peer evaluation methods, feedback structures, and the validity and reproducibility of peer evaluations, including inter-observer agreement and associations with other quality measures when available.

**Results:**

Of 2,135 retrieved references, 32 studies met inclusion criteria. Of these, 21 were from the field of gastroenterology, 5 from cardiology, 3 from pulmonology, and 3 from interventional radiology. Overall, 22 studies described the development or testing of peer scoring systems and 18 reported inter-observer agreement, which was good or excellent in all but 2 studies. Only 4 studies, all from gastroenterology, tested the association of scoring systems with other quality measures, and no studies tested the impact of peer evaluation on patient outcomes. Best practices included standardized scoring systems, prospective criteria for case selection, and collaborative and non-judgmental review.

**Conclusions:**

Peer evaluation of invasive medical procedures is feasible and generally demonstrates good or excellent inter-observer agreement when performed with structured tools. Our review identifies common elements of successful interventions across specialties. However, there is limited evidence that peer-evaluated performance is linked to other quality measures or that feedback to clinicians improves patient care or outcomes. Additional research is needed to develop and test peer evaluation and feedback interventions.

**Supplementary Information:**

The online version contains supplementary material available at 10.1186/s12909-022-03652-9.

## Introduction

Invasive medical procedures such as endoscopy, percutaneous coronary intervention (PCI), and bronchoscopy are highly effective for the diagnosis and treatment of disease when used appropriately [1–3]. However, variability in operator performance of these procedures has been widely reported, sometimes resulting in suboptimal procedural outcomes or patient harm [4–7]. Clinical societies therefore recommend standardized processes to assess clinician competency and to monitor care quality and outcomes [2, 8, 9].

Peer evaluation is one common mechanism for assessing procedural quality and providing meaningful feedback to physicians. Multiple formats have been described, including Morbidity and Mortality (M&M) conference, root cause analysis, and random case reviews. Peer review is mandated for some cardiac procedures [10], and clinicians perceive peer feedback to be highly useful [11, 12]. Among procedural training programs, structured evaluation and feedback is now ubiquitous and there are numerous tools to guide the evaluation of trainees [13–16]. However, there is little guidance on how to optimally implement a peer evaluation program among practicing clinicians after the completion of mandatory training.

Peer evaluation may be particularly useful for the assessment of procedures within the field of internal medicine. These procedures can generate a durable record (photo, video, or angiography) and involve both clinical decision-making and technical performance. Since there is limited literature on this topic for any single procedure or subspecialty, we sought to review studies among all internal medicine procedural subspecialties and related specialties that use percutaneous or minimally invasive techniques, including interventional radiology and vascular surgery. We hypothesized that some characteristics of successful peer evaluation programs may be common among all invasive medical procedures. We therefore performed a systematic review to: 1) identify and characterize peer evaluation tools for practicing procedural clinicians; 2) assess evidence for the validity of peer evaluations; and 3) describe best practices of peer evaluation programs.

## Methods

We conducted a systematic review according to the Preferred Reporting Items for Systematic Reviews and Meta-analyses (PRISMA) recommendations [17]. Our protocol is registered on the International Prospective Register of Systematic Reviews (PROSPERO) (CRD42020209345).

### Data sources and searches

We conducted a search of Medline and Embase from database inception through September 7, 2021 using a search strategy developed in consultation with a research librarian (Louden D). Search strategies (Appendix) incorporated controlled vocabulary terms and keywords appropriate to each database to represent the concepts of peer evaluation and peer feedback for procedures in the field of internal medicine and related subspecialties. Interventional radiology (IR) and endovascular surgical procedures were included since these commonly use percutaneous techniques similar to internal medicine subspecialty procedures. Reference lists of studies meeting the inclusion criteria were manually reviewed for additional articles.

### Study selection

We imported citations into Covidence (Melbourne, Australia). We included a study if it was a clinical trial or an observational study (prospective or retrospective) published in English that reported on peer assessment and/or peer feedback of internal medicine subspecialty, IR, or endovascular surgical procedures. We excluded a study if it reported only on trainee performance (medical students, residents, fellows) or only on the use of procedural simulators. Two reviewers (Doll JA, Thai TN) independently performed a title and abstract screen to identify potential citations for subsequent full-text review. Inter-reviewer discrepancies were resolved by consensus after full-text review by both reviewers. Included studies were reviewed with clinical content experts for appropriateness and completeness.

### Data extraction and study quality

A standardized data abstraction form was created to extract prespecified data points from each included study (Appendix). Two reviewers (Doll JA, Thai TN) independently extracted qualitative data from each reference, including study type, procedure evaluated, scoring system, presence of agreement testing, feedback structure and content, outcomes assessment, and assessment of overall study quality. Study quality was assessed using a scale modified from the Oxford Centre for Evidence-based Medicine [18, 19]. This scale rates studies from 1 to 5, with 1a as highest quality (systematic review of randomized controlled trials) and 5 as lowest quality (expert opinion). Differences in classification were resolved by consensus. The two reviewers jointly extracted quantitative data including number of procedures, number of evaluated clinicians, number or evaluators, and agreement testing results. We used the framework described by Messick to characterize evidence of validity for peer evaluation processes [20].

## Results

### Study selection

The review process is depicted in the PRISMA flow chart (Appendix Fig. 1). A total of 2,703 citations were identified initially by our electronic search strategy; 568 duplicates were removed for a total of 2,135 for review. Of these, 90 full-text articles were reviewed, and 23 studies met our inclusion/exclusion criteria. After review of references of these articles, we included an additional 9 studies. The final sample of 32 studies included 21 from the subspecialty of gastroenterology [21–41], 5 from cardiology [42–46], 3 from pulmonology [47–49], and 3 from IR [50–52] (Table 1).

**Table 1:** Studies of peer evaluation for invasive medical procedures

Subspecialty	Source	Procedure	Research Design	Evaluation Method	Feedback Method	Study Quality*
Cardiology	Blows 2012	PCI	Retrospective	Angiography and PCI review	Benchmarked report	4
	Doll 2017	PCI	Retrospective	Case conference	M&M conference	4
	Doll 2019	Coronary Angiography and PCI	Retrospective	Angiography and clinical record review	M&M conference	4
	Puri 2016	PCI	Retrospective	Clinical record review	None	4
	Rader 2014	Coronary Angiography	Prospective, agreement testing	Video review	None	2b
Gastroenterology	Barton 2012	Colonoscopy	Prospective, agreement testing	Live, in-person review	None	2b
	Boyle 2012	Colonoscopy	Prospective	Live, in-person review	None	2b
	Duloy 2018	Polypectomy	Prospective, agreement testing	Video review	None	2b
	Duloy 2019	Polypectomy	Prospective, agreement testing	Video review	Report card	2b
	Fleischer 1992	Various endoscopies	Retrospective	Case conference	M&M conference	4
	Gupta 2011	Polypectomy	Prospective, agreement testing	Video review	None	2b
	Gupta 2012	Polypectomy	Prospective, agreement testing	Video review	None	2b
	Keswani 2020	Colonoscopy	Prospective, agreement testing	Video review	None	2b
	Lee 2011	Colonoscopy	Prospective, agreement testing	Video review	None	2b
	Mai 1991	Colonoscopy, endoscopies	Retrospective	Clinical record review	Monthly Meeting	4
	Patel 2019	Cold-snare polypectomy	Prospective, agreement testing	Video review	None	2b
	Rex 2000	Colonoscopy	Prospective, agreement testing	Video review	None	3b
	Rex 2010	Colonoscopy	Prospective	Video review	None	2b
	Sapienza 1992	Colonoscopy, endoscopic gastrostomy, sigmoidoscopy, and upper endoscopy	Retrospective	Case conference	Letter	4
	Sarker 2008	Colonoscopy and flexible sigmoidoscopy	Prospective, agreement testing	Live, in-person review	None	2b
	Scaffidi 2018	Colonoscopy	Prospective, agreement testing	Live, in-person review (non-blinded) and retrospective video review (blinded)	None	2b
	Shah 2002	Colonoscopy	Prospective, agreement testing	Video review	None	2b
	Takao 2020	ESD	Prospective, agreement testing	Video review	None	2b
	Thomas-Gibson 2006	Flexible sigmoidoscopy	Prospective, agreement testing	Video review	None	2b
	Vassiliou 2010	Colonoscopy, upper endoscopy	Prospective, agreement testing	Live, in-person review	None	2b
	Walsh 2015	Colonoscopy	Prospective, agreement testing	Live, in-person review	None	2b
Interventional Radiology	Caruso 2016	Various IR procedures	Retrospective	Multi-component review	M&M conference	4
	d'Othée 2013	Various IR procedures	Retrospective	Case conference	Daily conference	4
	Luo 2019	Various IR procedures	Retrospective	Case conference	Daily conference	4
Pulmonology	Konge 2012 (1)	Bronchoscopy	Prospective, agreement testing	Video review	None	2b
	Konge 2012 (2)	FNA guided by EUS	Prospective, agreement testing	Direct observation & video review	None	2b
	Konge 2015	EBUS-TBNA	Prospective, agreement testing	Video review	None	2b

^*^Rating scale from the Oxford Center for Evidence-based Medicine [18]

Abbreviations: *PCI* Percutaneous coronary intervention, *M&M* Morbidity & Mortality, *ESD* Endoscopic submucosal dissection, *IR* Interventional radiology, *FNA* Fine-needle aspiration, *EUS* Endoscopic ultrasound, *EBUS-TBNA* Endobronchial ultrasound-guided transbronchial needle aspiration

### Peer evaluation and feedback processes

The studies reported peer evaluation using various methods or a combination of multiple methods: review of video or fluoroscopy recordings, in-person observation, and review of medical records. For gastroenterology procedures, most studies used retrospective review of videos. Shah et al. provided simultaneous recording of the endoscopists' hands in addition to the endoscopic intraluminal view and colonoscope configuration [34]. Most other gastroenterology studies provided the endoscopic view only, and some selectively edited the videos to concentrate on a specific task, typically a polypectomy. For cardiology studies, Rader et al. created a video of coronary angiography procedures that included a case description and views of the operators’ hands and the fluoroscopy images [46]. Other cardiology studies included review of case records with the fluoroscopy images. The 3 pulmonology studies utilized endobronchial videos with associated ultrasound videos where appropriate [47–49]. IR reviews were performed collectively in a group setting by review of medical history and procedural details [50–52]. A scoring system for peer evaluation was developed or tested in 22 of the studies (Table 2) [21, 22, 24–28, 30, 33–41, 44, 46–49]. These scoring systems commonly included assessment of technical skills and clinical decision-making.

**Table 2:** Studies that develop or test scoring systems for peer evaluation of invasive medical procedure performance

Scoring System (Procedure)	Source	Agreement testing	Number of Evaluators	Number of Clinicians Evaluated	Total Cases Reviewed
CARS (Coronary Angiography)	Rader 2014	ICC = 0.42 and 0.49	2	10	40
CIQ (Colonoscopy)	Keswani 2020	Spearman r = 0.92	9	17	102
	Lee 2011	None	4	11	110
	Rex 2000	None	4	2	20
CSPAT (Cold-snare Polypectomy)	Patel 2019	Kappa = 0.58 (overall)	13	Not specified	55
DOPS (Colonoscopy)	Barton 2012	G-theory analysis: G = 0.81	28	147	162
DOPyS (Polypectomy)	Duloy 2018	Weighted Cohen's k = 0.80	2	13	130
	Duloy 2019	Weighted Cohen's k = 0.69	2	11	220
	Gupta 2011	G-theory analysis	7	23	60
	Gupta 2012	Kappa = 0.32 (trained assessors), Kappa = 0.07 (untrained assessors)	4	8	32
EBUSAT (EBUS-TBNA)	Konge 2015	Cronbach's alpha = 0.95	3	18	58
EVAT (Endoscopic Submucosal Dissection)	Takao 2020	ICC = 0.87	3	8	17
GAGES (Colonoscopy, Upper Endoscopy)	Vassiliou 2010	ICC 0.97 for GAGES-C and 0.96 for GAGES-UE	Not specified	139	139
GiECAT (Colonoscopy)	Scaffidi 2018	ICC = 0.91 (inter-rater), 0.85 (live vs. video)	7	40	40
	Walsh 2015	ICC = 0.85	Not specified	61	116
Unnamed (Percutaneous Coronary Intervention)	Blows 2012	None	3	10	326
Unnamed (Colonoscopy)	Boyle 2012	None	Not specified	8	24
Unnamed (Bronchoscopy)	Konge 2012 (1)	Cronbach's alpha = 0.86	2	19	57
Unnamed (FNA guided by EUS)	Konge 2012 (2)	Cronbach's alpha = 0.93	3	6	30
Unnamed (Colonoscopy and flexible sigmoidoscopy)	Sarker 2008	Cronbach’s alpha = 0.80 and 0.83	Not specified	9	77
Unnamed (Colonoscopy)	Shah 2002	Kappa = 0.63 (overall)	3	18	22
Unnamed (Flexible Sigmoidoscopy)	Thomas-Gibson 2006	ICC = 0.89	Not specified	Not specified	43

Abbreviations: *CARS* Coronary angiography rating scale, *CIQ* Colonoscopy inspection quality, *CSPAT* Cold-snare polypectomy assessment tool, *DOPS* Direct Observation of Procedural Skills, *DOPyS* Direct Observation of Polypectomy Skills, *EBUSAT* Endobronchial ultrasound assessment tool, *EBUS-TBNA* Endobronchial ultrasound-guided transbronchial needle aspiration, *EVAT* ESD video assessment tool, *ESD* Endoscopic submucosal dissection, *GAGES* Global Assessment of Gastrointestinal Endoscopic Skills, *GiECAT* Gastrointestinal Endoscopy Competency Assessment Tool, *FNA* Fine-needle aspiration, *EUS* Endoscopic ultrasound, *ICC* Intraclass correlation coefficient

Feedback to clinicians was described in 10 studies [22, 23, 28, 32, 42–44, 50–52]. Feedback methods included personalized score cards, letters from review committees, and group discussion during case conferences. In Blows et al., each clinician was given a feedback report, benchmarked against peers, that included assessment of anatomical suitability for PCI, lesion severity, appropriateness of intervention strategy, and satisfactory outcome [44]. Caruso et al. describe a two-tiered process for IR reviews [50]. An initial review of random cases by peer radiologists would trigger a group discussion at M&M conference if any concerns about clinical management are identified.

### Validity evidence

Inter-observer agreement of peer evaluations was tested in 18 of the studies [21, 22, 24–26, 29, 33–39, 41, 46–49], using various statistical methodologies including Cohen’s kappa, Cronbach’s alpha, intraclass correlation coefficient (ICC), Spearman correlation, and the generalizability theory (G-theory) (Table 2). All but two studies [25, 46] demonstrated at least a moderate degree of agreement between observers, with most studies revealing good or excellent agreement (Table 2). Most studies described training on the use of the assessment instrument, and Gupta et al. demonstrated that assessors without training were unable to differentiate between expert and non-expert endoscopists [25]. Of the inter-observer agreement studies, six [24, 36, 40, 46–48] calculated the minimum number of observations required to reliably evaluate an operator. These estimates ranged from 1 assessor evaluating 3 procedures [47] to 3 assessors rating 7 procedures [46] to reach at least moderate degree of agreement.

Fifteen studies [25–27, 30, 33–38, 40, 41, 46–49] tested the relationship of peer evaluation to other variables by assessing clinicians with varying expertise. More experienced clinicians performed better than less experienced clinicians. Gupta et al. demonstrated that assessors using the Direct Observation of Polypectomy Skills (DOPyS) instrument could reliably distinguish between the expert and intermediate endoscopists [21]. Similarly, Konge et al. demonstrated the Endoscopic Ultrasonography Assessment Tool (EU-SAT) discriminates between trainees and experienced physicians with regard to ultrasonographic fine needle aspiration; the experienced physicians not only performed better than the trainees, but performance assessments were also more consistent [39]. The only exception, Shah et al., did not find a significant difference among colonoscopists who performed 100, 250, 500, or 1000 prior colonoscopies [34].

Only 4 studies described the association of peer evaluation with other quality measures [21, 26, 27, 30]. Two studies of the Colonoscopy Inspection Quality (CIQ) tool [27, 30] demonstrated that peer evaluated technique was associated with adenoma detection rate (ADR), a key measure of quality since lower ADR is associated with increased risk of post-colonoscopy colorectal cancer [53]. Keswani et al. showed that novice CIQ scores significantly correlated with ADR and withdrawal time (WT); and novice proximal colon CIQ scores significantly correlated with serrated polyp detection rate [26]. However, Deloy et al. showed that polypectomy competency assessed by DOPyS did not correlate with the unrelated colonoscopy quality measures WT and ADR [21].

There were 6 studies [22, 28, 31, 32, 44, 45] that assessed the impact of peer evaluation on clinician performance. None of these had a randomized design. Prospective observational designs were used in 5 studies [22, 28, 31, 32, 44] to measure clinician performance before and after implementation of a peer evaluation intervention. In Duloy et al., feedback was given in the form of a personalized polypectomy skills report card [22]. The mean performance score of polyps removed significantly increased in the post–report card phase. Four studies [28, 32, 44, 45] provided feedback regarding case selection and procedural appropriateness; each demonstrated a decline in inappropriate procedures after the feedback period. In one study [31], clinician knowledge that they were being observed via videotaping (without receiving feedback) was associated with increased colonoscopy inspection time and improved measures of mucosal inspection technique. There were no studies linking peer evaluation and feedback to patient outcomes.

### Best practices for implementation of peer evaluation

Finally, 6 studies [23, 42, 43, 50–52] described best practices for peer evaluation interventions without providing specific evidence of validity. Common elements included pre-specified criteria for case selection, a protected and non-punitive environment, and a focus on education and quality improvement. Doll et al. described a national peer review committee for PCI complications that provided operators with an overall rating and recommendations for improvement [43]. Luo et al. proposed that peer review in a group setting allows the operator an opportunity to provide context and rationale for clinical management [52]. All studies recommended routine, transparent processes that are applied to all clinicians in the group.

## Discussion

This systematic review shows that peer evaluation for invasive medical procedures is feasible and has considerable evidence of validity, primarily based on studies reporting excellent inter-observer agreement. No randomized studies are available and there are limited studies demonstrating an association of peer-evaluated performance with other quality measures or patient outcomes. Additional research is needed to develop and test peer evaluation and feedback interventions, particularly using randomized designs and with meaningful clinical outcomes. However, this review identifies common elements of successful interventions across specialties and provides a template for hospitals or health systems seeking to establish or refine peer evaluation programs.

The importance of peer evaluation for proceduralists has been established since at least the 1990s [54, 55]. Innovations in peer evaluation have been traditionally led by the surgical and anesthesiology communities, including the creation of the M&M conference that is now ubiquitous among both surgery and internal medicine training programs [56]. Surgeons have also outpaced the internal medicine sub-specialties in the validation of peer evaluation methods—17 unique tools are available for assessment of laparoscopic cholecystectomy, for example [57]—and providing feedback and training interventions to improve performance [58]. Since the literature examining any specific procedure within the internal medicine subspecialities is limited, and since these procedures share many common characteristics, our review examines the validity and best practices of peer evaluation across multiple related procedures, including percutaneous procedures in IR.

Using the validity framework established by Messick and others [20], our review highlights substantial evidence of *content*, *internal structure,* and *relationship to other variables* sources of validity. Evaluation methods were typically developed by clinicians and utilized observation of performance either directly or via durable medical media such as videos. Inter-observer agreement was high for most tools. Evaluated performance mostly correlated to objective measures of experience such as level of training or number of procedures performed. However, the *consequences* source of validity was notably lacking since studies were not designed or powered to establish impacts on clinician performance or patient outcomes. In addition, studies variably reported *response process* information, and characteristics of scoring systems varied widely. Therefore, it is unclear if existing evaluative tools are optimized for clinical practice. Validity evidence is strongest for assessment of endoscopic and bronchoscopic procedures, and lacking or of low quality for some cardiac, pulmonary, and IR procedures.

For now, groups seeking to establish peer evaluation programs should use a tool with validity evidence when available (Table 2). Existing scores share common elements. Performance is typically summarized across multiple domains with numerical values, often including a pre-specified threshold for competency. For example, for the Coronary Angiography Rating Scale (CARS), Rader et al. used an assessment form with 29 items to be scored on a scale of 1 to 5, and a summary score presented on a scale of 1 to 9 [46]. Similarly, for DOPyS (polypectomy), Gupta et al. describe a 33-point structured checklist and global assessment using a 1 to 4 scale [24]. These scores can provide feedback on specific components of the procedure under the direct control of the operator such as case selection/appropriateness, strategy and decision-making, technical skills, outcomes, and documentation, as well as an overall summary of performance. Since scoring systems are lacking for many procedures, clinical groups may consider adapting and testing scores from other procedures to meet their individual needs.

The optimal evaluative method will depend on institutional goals and resources. Direct observation of performance, for example, has the advantage of real-time assessment and visualization of all aspects of the procedure. Its disadvantages include lack of blinding/anonymity, substantial time burden for the assessor, and the potential for bias. Conversely, post hoc review of reports and images may be more objective and efficient, but may miss important procedural details or environment factors outside the control of the observed proceduralist.

Our review identified two general types of peer feedback programs. Group-based, collaborative peer review in the setting of M&M or case review conferences is recommended for non-judgmental, educational discussions. Cases are triggered for review by complications, poor patient outcomes, or high educational content. Alternatively, anonymous or blinded review may be more appropriate for quality surveillance, sometimes with random case selection. Individualized feedback to clinicians may identify opportunities for practice improvement.

Most included studies reported peer evaluation and feedback activities in the context of education and quality improvement programs. However, there may also be a role for peer evaluation for quality assessment or recertification for practice. In the United States, the Joint Commission on Accreditation of Healthcare Organization (JCAHO) requires assessment of clinician performance to obtain or retain hospital credentials (via Ongoing Professional Practice Evaluations (OPPE) and Focused Professional Practice Evaluations (FPPE)) [59]. Other countries and health systems use similar structures to ensure clinical competence and promote lifetime learning [60]. Standardized methods and scoring systems could enhance these efforts. For endoscopic gastroenterology procedures, there is potential for current peer assessment tools to be utilized as part of a standardized competency assessment [61]. However, this strategy has yet to be tested, and additional research is required to establish appropriate thresholds for clinician competency and excellence. Achieving widespread dissemination of these tools may require support from clinical societies and health systems, since clinicians will require support and resources to learn and apply these methods.

Our systematic review has several limitations that merit discussion. Only English language studies were reviewed. We excluded studies that solely examined trainee evaluation. While our aim was to examine peer evaluation of practicing clinicians, it is possible that some tools developed for trainees could also be useful in this setting. We found marked heterogeneity in the design of the included studies, and many were of low quality. This precluded meta-analysis of results. Many studies did not include a formal scoring system, and those that did used differing testing methods to assess validity. Some elements of successful peer evaluation may be highly specific to individual procedures. Our attempt to generalize across multiple invasive procedures may therefore miss important nuances that are highlighted by the procedure-specific studies. Finally, though our search strategy included procedure-specific terminology (i.e. “colonoscopy”) and more general terms (i.e. “endovascular procedure”) it is possible that our search was biased towards certain procedures and omitted important studies. However, review of reference lists from included studies did not reveal a significant body of literature missed by our search strategy.

## Conclusion

Our systematic review describes common elements of peer evaluation and feedback interventions for a subset of invasive medical procedures. Peer evaluation is a feasible and reproducible method of assessing practicing procedural physicianss. However, there are limited data on the relationship of peer evaluation to other quality measures and patient outcomes. Additional research is needed, ideally in the form of prospective and randomized outcomes studies evaluating the impact of peer evaluation on clinician performance and patient outcomes.

## Supplementary Information

**Additional file 1.** 

## Data Availability

The datasets used and/or analyzed during this study are available from the corresponding author on reasonable request.
