# Supplementary material for: Peer evaluation and feedback for invasive medical procedures: a systematic review
Source: BMC Med Educ. 2022 Jul 29;22:581. doi: 10.1186/s12909-022-03652-9 (PMC9335975; doi:10.1186/s12909-022-03652-9)
Supplement: Supplementary file 1 — Additional file 1. [file 12909_2022_3652_MOESM1_ESM.docx]

Figure 1. PRISMA Flowchart Outlining the Literature Search and Article Evaluation Process

**Identification of studies via databases and registers**

Records identified from Medline and Embase through

September 7, 2021 (n = 2703)

Records removed *before screening*:

Duplicate records removed

(n = 568)

**Identification**

Records after duplicates removed (n = 2135)

Records excluded after title and abstract review (n = 2045)

Records excluded:

Ineligible populations (n = 10)

Non-peer feedback (n = 14)

Not a report of a new discovery (n = 25)

Not an invasive procedure (n = 18)

Full-text articles assessed for eligibility (n =90)

**Screening**

Records that fulfilled eligibility criteria (n = 23)

Additional records from reference review (n = 9)

Studies included in review

(n = 32)

**Included**

**Supplemental Text: Search strategies for Medline and Embase**

**Medline (Ebsco) – 922 references limited to English – 9/7/21**

MH ("Peer Review, Health Care+" OR "Professional Review Organizations+" OR “Root Cause Analysis+” OR (“Peer Review” NOT (“Editorial Policies+” OR “Periodicals as Topic+” OR “Research Standards+”)) OR “Formative Feedback+” OR (“Clinical Competence+” AND “Video Recording+”)) OR TI ("peer review" OR “peer reviews” OR "peer learning" OR "peer feedback" OR "peer evaluation" OR “case review” OR “case reviews” OR “case review conference” OR “case review conferences” OR "peer assessment" OR “peer assessments” OR “peer assisted” OR “peer teaching” OR “peer rating” OR “peer ratings” OR “peer to peer” OR “peer audit” OR “peer audits” OR “peer physicians” OR “physician peers” OR “peer surgeons” OR “surgeon peers” OR “fellow surgeons” OR “fellow physicians” OR “peer group” OR “peer groups” OR “medical colleagues” OR "mortality conference" OR “mortality conferences” OR “multi source assessment” OR “multi source assessments” OR “multi source feedback” OR “multi source evaluation” OR “multi source evaluations” OR “multisource assessment” OR “multisource assessments” OR “multisource feedback” OR “multisource evaluation” OR “multisource evaluations” OR “360 assessment” OR “360 assessments” OR “Root cause analysis” OR “root cause analyses” OR (audit N2 feedback) OR (trigger* N3 review) OR (trigger* N3 reviews) OR “review committee” OR “review committees” OR “review panel” OR “review panels” OR ((peer OR peers) N5 (rating* OR review OR reviews OR feedback OR evaluation* OR assessment*)) OR coach OR coaching OR (“video based” AND (assess* OR evaluat* OR teach* OR rating* OR review* OR feedback OR audit*)) OR “video review” OR “video reviews” OR “video rating” OR “video ratings” OR “surgical audit” OR “surgical audits” OR “procedural audit” OR “procedural audits” OR “procedure audit” OR “procedure audits” OR ((“positive feedback” OR “negative feedback”) NOT “feedback loop” NOT (MH “Animals” NOT MH “Humans”)) OR “formative feedback” OR “constructive feedback” OR “meaningful feedback”) OR AB ("peer review" OR “peer reviews” OR "peer learning" OR "peer feedback" OR "peer evaluation" OR “case review conference” OR “case review conferences” OR "peer assessment" OR “peer assessments” OR “peer assisted” OR “peer teaching” OR “peer rating” OR “peer ratings” OR “peer to peer” OR “peer audit” OR “peer audits” OR “peer physicians” OR “physician peers” OR “peer surgeons” OR “surgeon peers” OR “fellow surgeons” OR “fellow physicians” OR “peer group” OR “peer groups” OR “medical colleagues” OR "mortality conference" OR “mortality conferences” OR “multi source assessment” OR “multi source assessments” OR “multi source feedback” OR “multi source evaluation” OR “multi source evaluations” OR “multisource assessment” OR “multisource assessments” OR “multisource feedback” OR “multisource evaluation” OR “multisource evaluations” OR “360 assessment” OR “360 assessments” OR “Root cause analysis” OR “root cause analyses” OR (audit N2 feedback) OR (trigger* N3 review) OR (trigger* N3 reviews) OR “review committee” OR “review committees” OR “review panel” OR “review panels” OR ((peer OR peers) N5 (rating* OR review OR reviews OR feedback OR evaluation* OR assessment*)) OR coach OR coaching OR (“video based” AND (assess* OR evaluat* OR teach* OR rating* OR review* OR feedback OR audit*)) OR “video review” OR “video reviews” OR “video rating” OR “video ratings” OR “surgical audit” OR “surgical audits” OR “procedural audit” OR “procedural audits” OR ((“positive feedback” OR “negative feedback”) NOT “feedback loop” NOT (MH “Animals” NOT MH “Humans”)) OR “procedure audit” OR “procedure audits” OR “formative feedback” OR “constructive feedback” OR “meaningful feedback”)

AND

MH ("Percutaneous Coronary Intervention+" OR "Angioplasty, Balloon, Coronary+" OR "Atherectomy, Coronary+" OR "Cardiac Catheterization+" OR "Cardiac Catheters+" OR "Coronary Angiography+" OR "Angiocardiography+" OR "Cardiac Electrophysiology+" OR "Electrophysiologic Techniques, Cardiac+" OR "Catheter Ablation+") OR TI (“interventional cardiology” OR “invasive cardiac” OR “percutaneous coronary” OR “coronary intervention” OR “coronary interventions” OR angiocardiograph* OR “coronary angiograms”) OR AB (“interventional cardiology” OR “invasive cardiac” OR “percutaneous coronary” OR “coronary intervention” OR “coronary interventions” OR angiocardiograph* OR “coronary angiograms”) OR TI ((stent OR stents OR stenting OR catheter* OR angioplast* OR angiograph* OR atherectom* OR electrophysiolog* OR ablate* OR ablation) N3 (coronary OR cardiac OR intracardiac OR heart)) OR AB ((stent OR stents OR stenting OR catheter* OR angioplast* OR angiograph* OR atherectom* OR electrophysiolog* OR ablate* OR ablation) N3 (coronary OR cardiac OR intracardiac OR heart))

OR

MH ("Vascular Surgical Procedures+" OR "Endovascular Procedures+" OR "Catheterization, Peripheral+" OR "Radiography, Interventional+" OR "Radiology, Interventional+") OR TI (“endovascular procedure” OR “endovascular procedures” OR “percutaneous treatment” OR “percutaneous treatments” OR “percutaneous intervention” OR “percutaneous interventions” OR Endarterectom* OR embolectom* OR atherectom* OR thrombectom* OR “interventional radiology” OR “interventional radiography” OR angioplast* OR “peripheral catheterization” OR picc OR “central venous catheterization” OR “central catheterization” OR ((Endovascular OR intravascular) N3 (graft* OR repair* OR treatment* OR intervention* OR procedure* OR technique* OR surgery OR surgeries OR surgical*)) OR (Vascular N3 (surgery OR surgeries OR surgical* OR graft* OR repair*))) OR AB (“endovascular procedure” OR “endovascular procedures” OR “percutaneous treatment” OR “percutaneous treatments” OR “percutaneous intervention” OR “percutaneous interventions” OR Endarterectom* OR embolectom* OR atherectom* OR thrombectom* OR “interventional radiology” OR “interventional radiography” OR angioplast* OR “peripheral catheterization” OR picc OR “central venous catheterization” OR “central catheterization” OR ((Endovascular OR intravascular) N3 (graft* OR repair* OR treatment* OR intervention* OR procedure* OR technique* OR surgery OR surgeries OR surgical*)) OR (Vascular N3 (surgery OR surgeries OR surgical* OR graft* OR repair*)))

OR

(MH ("Endoscopy, Gastrointestinal+" OR "Colonoscopy+” OR "Sigmoidoscopy+” OR "Bronchoscopy+” OR "Thoracentesis+” OR "Paracentesis+” OR "Tracheostomy+” OR "Nephrostomy, Percutaneous+” OR "Nephrotomy+” OR "Nephrolithotomy, Percutaneous+” OR "Cholangiopancreatography, Endoscopic Retrograde+” OR "Endoscopy, Digestive System+” OR "Gastrostomy+”) OR TI (colonoscop* OR sigmoidoscop* OR bronchoscop* OR thoracentesis OR tracheostom* OR paracentesis OR nephrostom* OR nephrolithotom* OR cholangiopancreatography* OR ERCP OR esophagogastroduodenoscop* OR gastrostom* OR Esophagoscop* OR gastroscop* OR endoscop*) OR AB (colonoscop* OR sigmoidoscop* OR bronchoscop* OR thoracentesis OR tracheostom* OR paracentesis OR nephrostom* OR nephrolithotom* OR cholangiopancreatography* OR ERCP OR esophagogastroduodenoscop* OR gastrostom* OR Esophagoscop* OR gastroscop* OR endoscop*))

**Embase (Elsevier) – 1781 references limited to English – 9/7/21**

'peer review organization'/exp OR 'professional standards review organization'/exp OR ('peer review'/exp NOT ('publication'/exp OR 'publishing'/exp)) OR 'root cause analysis'/exp OR 'constructive feedback'/exp OR ('clinical competence'/exp AND 'videorecording'/exp) OR ("peer review" OR “peer reviews” OR "peer learning" OR "peer feedback" OR "peer evaluation" OR “case review” OR “case reviews” OR “case review conference” OR “case review conferences” OR "peer assessment" OR “peer assessments” OR “peer assisted” OR “peer teaching” OR “peer rating” OR “peer ratings” OR “peer to peer” OR “peer audit” OR “peer audits” OR “peer physicians” OR “physician peers” OR “peer surgeons” OR “surgeon peers” OR “fellow surgeons” OR “fellow physicians” OR “peer group” OR “peer groups” OR “medical colleagues” OR "mortality conference" OR “mortality conferences” OR “multi source assessment” OR “multi source assessments” OR “multi source feedback” OR “multi source evaluation” OR “multi source evaluations” OR “multisource assessment” OR “multisource assessments” OR “multisource feedback” OR “multisource evaluation” OR “multisource evaluations” OR “360 assessment” OR “360 assessments” OR “Root cause analysis” OR “root cause analyses” OR (audit NEAR/2 feedback) OR (trigger* NEAR/3 review) OR (trigger* NEAR/3 reviews) OR “review committee” OR “review committees” OR “review panel” OR “review panels” OR ((peer OR peers) NEAR/5 (rating* OR review OR reviews OR feedback OR evaluation* OR assessment*)) OR coach OR coaching OR (“video based” NEAR/3 (assess* OR evaluat* OR teach* OR rating* OR review* OR feedback OR audit*)) OR “video review” OR “video reviews” OR “video rating” OR “video ratings” OR “surgical audit” OR “surgical audits” OR “procedural audit” OR “procedural audits” OR “procedure audit” OR “procedure audits” OR “formative feedback” OR “constructive feedback” OR “meaningful feedback”):ti,ab

OR

('positive feedback':ti,ab OR 'negative feedback':ti,ab) NOT (‘feedback regulation’:ti,ab OR 'feedback loop':ti,ab) NOT (('nonhuman'/exp OR 'animal'/exp OR 'in vitro study'/exp) NOT 'human'/exp)

AND

('percutaneous coronary intervention'/exp OR 'interventional cardiovascular procedure'/exp OR 'atherectomy'/exp OR 'laser angioplasty'/exp OR 'percutaneous transluminal angioplasty'/exp OR 'transluminal coronary angioplasty'/exp OR 'heart catheterization'/exp OR 'heart catheter'/exp OR 'angiocardiography'/exp OR 'heart electrophysiology'/exp OR 'electrophysiological procedures'/exp OR 'catheter ablation'/exp OR (“interventional cardiology” OR “invasive cardiac” OR “percutaneous coronary” OR “coronary intervention” OR “coronary interventions” OR angiocardiograph* OR “coronary angiograms”):ti,ab OR ((stent OR stents OR stenting OR catheter* OR angioplast* OR angiograph* OR atherectom* OR electrophysiolog* OR ablate* OR ablation) NEAR/3 (coronary OR cardiac OR intracardiac OR heart)):ti,ab)

OR

('vascular surgery'/exp OR 'catheterization'/exp OR 'interventional radiology'/exp OR (“endovascular procedure” OR “endovascular procedures” OR “percutaneous treatment” OR “percutaneous treatments” OR “percutaneous intervention” OR “percutaneous interventions” OR Endarterectom* OR embolectom* OR atherectom* OR thrombectom* OR “interventional radiology” OR “interventional radiography” OR angioplast* OR “peripheral catheterization” OR picc OR “central venous catheterization” OR “central catheterization” OR ((Endovascular OR intravascular) NEAR/3 (graft* OR repair* OR treatment* OR intervention* OR procedure* OR technique* OR surgery OR surgeries OR surgical*)) OR (Vascular NEAR/3 (surgery OR surgeries OR surgical* OR graft* OR repair*))):ti,ab)

OR

('gastrointestinal endoscopy'/exp OR 'bronchoscopy'/exp OR 'thoracocentesis'/exp OR 'paracentesis'/exp OR 'tracheostomy'/exp OR 'nephrostomy'/exp OR 'nephrotomy'/exp OR 'nephrolithotomy'/exp OR 'endoscopic retrograde cholangiopancreatography'/exp OR 'digestive tract endoscopy'/exp OR 'gastrostomy'/exp OR (colonoscop* OR sigmoidoscop* OR bronchoscop* OR thoracentesis OR tracheostom* OR paracentesis OR nephrostom* OR nephrolithotom* OR cholangiopancreatography* OR ERCP OR esophagogastroduodenoscop* OR gastrostom* OR Esophagoscop* OR gastroscop* OR endoscop*):ti,ab)

NOT 'conference abstract'/it AND [english]/lim

Data abstraction form

Study Author/Date

Study type:

Randomized

Prospective

Retrospective

Other

Procedure type (i.e. colonoscopy, bronchoscopy, etc)

Number of observers/evaluators

Number of evaluated subjects

Total number of procedures reviewed

Evaluation method

Scoring system Y/N

Scoring system name (if applicable)

Agreement testing performed Y/N

Agreement testing result (i.e. Cohen’s kappa, ICC, etc)

Feedback structure/content (if applicable)

Comparison to other outcomes Y/N

Outcomes (if applicable)

Study quality (Oxford scale)

Summary findings
